# Case report: emergent endovascular treatment for carotid cavernous fistulas presenting as intracranial hemorrhage

**DOI:** 10.3389/fneur.2023.1133259

**Published:** 2023-04-18

**Authors:** Zhao-Liang Li, Ai-Lin Chen, Ying Chen, De-Hong Yang, Yu-Hui Wan, Yao Wu, Chun-Gang Dai, Qing Zhu

**Affiliations:** Department of Neurosurgery, Second Affiliated Hospital of Soochow University, Suzhou, China

**Keywords:** carotid cavernous fistula, treatment, endovascular, intracranial hemorrhage, case report

## Abstract

**Objectives:**

This study aimed to discuss the clinical characteristics and emergent endovascular treatment of carotid cavernous fistulas presenting as intracranial hemorrhage.

**Methods:**

The clinical data of five patients with carotid cavernous fistulas, who presented with intracranial hemorrhage and who were admitted from January 2010 to April 2017, were analyzed retrospectively, and the diagnoses were confirmed by head computed tomography. Digital subtraction angiography was carried out in all the patients for the diagnosis and further emergent endovascular procedures. All patients were followed up to assess the clinical outcomes.

**Results:**

In total, five patients harbored five mono-lateral lesions; two of them were obliterated by detachable balloons, two by detachable coils, and one by detachable coils and Onyx glue. Only one patient was cured by another detachable balloon in the second session, and the other four patients were cured in the first session. At the 3- to 10-year follow-up, there was no intracranial re-hemorrhage in any of the patients; there was no recurrence of symptoms; and delayed occlusion of the parent artery was noted in one case.

**Conclusion:**

Emergent endovascular therapy is indicated for carotid cavernous fistulas presenting as intracranial hemorrhage. Individualized treatment according to the characteristics of different lesions is safe and effective.

## 1. Introduction

Carotid cavernous fistula (CCF) is a pathological shunt that originates between a high-pressure internal carotid artery and a low-pressure cavernous venous system. The clinical presentation depends on hyperemia in the veins around the cavernous sinus (1). The most common clinical manifestations are pulsating exophthalmos, conjunctival edema, and cranial nerve palsy. Consequently, intracranial hemorrhage (ICH) is relatively rare, but it is a critical event and can result in a worse prognosis (2). Since January 2010, five cases of CCF presenting as intracranial hemorrhage have been treated in our department. All of these patients were treated endovascularly with an emergent procedure and had a good prognosis.

## 2. Case report

### 2.1. Clinical data

From January 2010 to April 2017, five adult patients who harbored CCF presented with ICH and were admitted to our hospital; all of them had a history of head trauma. ICH was found in one patient after surgery for traumatic brain contusion, in two patients after embolization by detachable balloons (Goldbal2, Balt, France), and in another two patients who did not have any medical intervention. The duration from head trauma to ICH varied from 1 month to 20 years. All patients suffered from headaches on admission, where three of them had additional pulsatile exophthalmos, and two of them had conjunctival edema as well. No cranial nerve palsy was noted (Table 1).

**Table 1:** Clinical characteristics of patients.

	**Case 1**	**Case 2**	**Case 3**	**Case 4**	**Case 5**
**Gender**	**Male**	**Female**	**Female**	**Male**	**Female**
Age	33	39	65	48	64
Symptoms	Headache pulsatile exophthalmos	Headache pulsatile exophthalmos	Headache conjunctival edema	Headache pulsatile exophthalmos	Headache conjunctival edema
Time lag between presentation to intervention (hours)	1	1.5	1	0.5	2
Past history	Head trauma	Head trauma	Head trauma; Surgery for traumatic brain contusion	Head trauma	Head trauma
Reoperation		Endovascular embolization by detachable balloons		Endovascular embolization by detachable balloons	
Postoperative length of hospital stay(days)	6	7	12	14	5

### 2.2. Imaging data

All patients were diagnosed with ICH by emergency head computed tomography (CT), and there were two patients with intraparenchymal hemorrhage (IPH), one patient with subarachnoid hemorrhage (SAH), and two patients with IPH with SAH. All of the patients underwent emergency digital subtraction angiography (DSA) for the diagnosis of CCF. In the study, five mono-lateral direct CCFs with high flow (Barrow type A) were noted, and all fistulas were located in the cavernous segment (C4) of the internal carotid artery; two of them showed signs of total flow steal. Reflux drainage of cortical vein reflux was detected in all patients, including three patients that had reflux drainage to the straight sinus and four patients who had it to the superior sagittal sinus. Three of them had bulbous dilation of the drainage vein (s).

### 2.3. Endovascular therapy

Endovascular treatment by detachable balloon: The patients were placed in the supine position, and local anesthesia was given. The femoral artery approach was established by an 8F (French) sheath followed by the introduction of an 8F guiding catheter into the lesional ICA. Subsequently, a Goldbal2 balloon equipped at the tip of a MABDTE microcatheter (Balt, France) was navigated into the cavernous sinus through the fistula by the arterial flow. Then, the balloon was gradually dilated by filling it with diluting contrast agent (normal saline: ousu [iohyanol 300 mL, Yangzijiang Pharmaceutical Group Co., LTD., China] =1:1) until the fistula had disappeared angiographically. Finally, the balloon was detached *in situ* after control angiography, by which the abnormal shunt disappeared. If the cavernous sinus cavity was too large, an additional balloon was used.

Endovascular treatment by a detachable coil. The patients were placed in the supine position with general anesthesia. The femoral artery approach was established by a 6F sheath, followed by an introduction of a 6F guiding catheter into the lesional ICA. Under fluoroscopic monitoring in the roadmap mode, two Echelon 10 microcatheters (eV3, USA) were navigated by a Traxcess 14 microguidewire (MicroVention, USA) into the cavernous sinus through the fistula successively. Thereafter, a series of detachable coils (Axium, eV3, USA) was introduced to occlude the fistulae. After confirmation of the obliteration of the abnormal shunt, the coils were detached, followed by retreatment with a microcatheter. If necessary, a HyperGlide 4 × 20 balloon (eV3, USA) was navigated to cover the fistula in the ICA by an X-pedion 10 microguidewire (eV3, USA). Based on the complete occlusion of the ICA by dilation of the undetachable balloon, Onyx 18 glue (eV3, USA) was injected slowly under fluoroscopy monitoring. Serial control angiography was carried out after the deflation of the balloon until the abnormal shunt had disappeared. Thereafter, the balloon and microcatheters were removed.

### 2.4. Follow-up

Scheduled head CT was performed immediately after the endovascular procedure, on the 2nd day after treatment, and on the day of discharge. The modified Rankin Scale (mRS) score for each of the patients was evaluated when they were discharged. All patients were followed up in the outpatient setting to detect any novel neurological deficits. The patients underwent follow-up CTs at 1 month, 3 months, 6 months, and 1 year after the endovascular procedure, and follow-up DSAs were performed at 6 months and 1 year after the procedure.

## 3. Results

A total of five patients (harboring 5 CCFs) underwent six endovascular procedures; two of them were cured by detachable balloons (one patient by two balloons and another by three balloons), two patients by detachable coils, and one patient by Onyx glue and detachable coils. Among them, only one patient was cured in a second session by detachable balloons, and the other four patients were cured in one session; two patients who were treated with detachable balloons experienced transient headaches after the procedure, which were spontaneously alleviated by medication. There were no novel neurological deficits immediately after the endovascular procedures. The mRS score at discharge was 0 in four patients and 1 in one patient.

**Figure 1:**
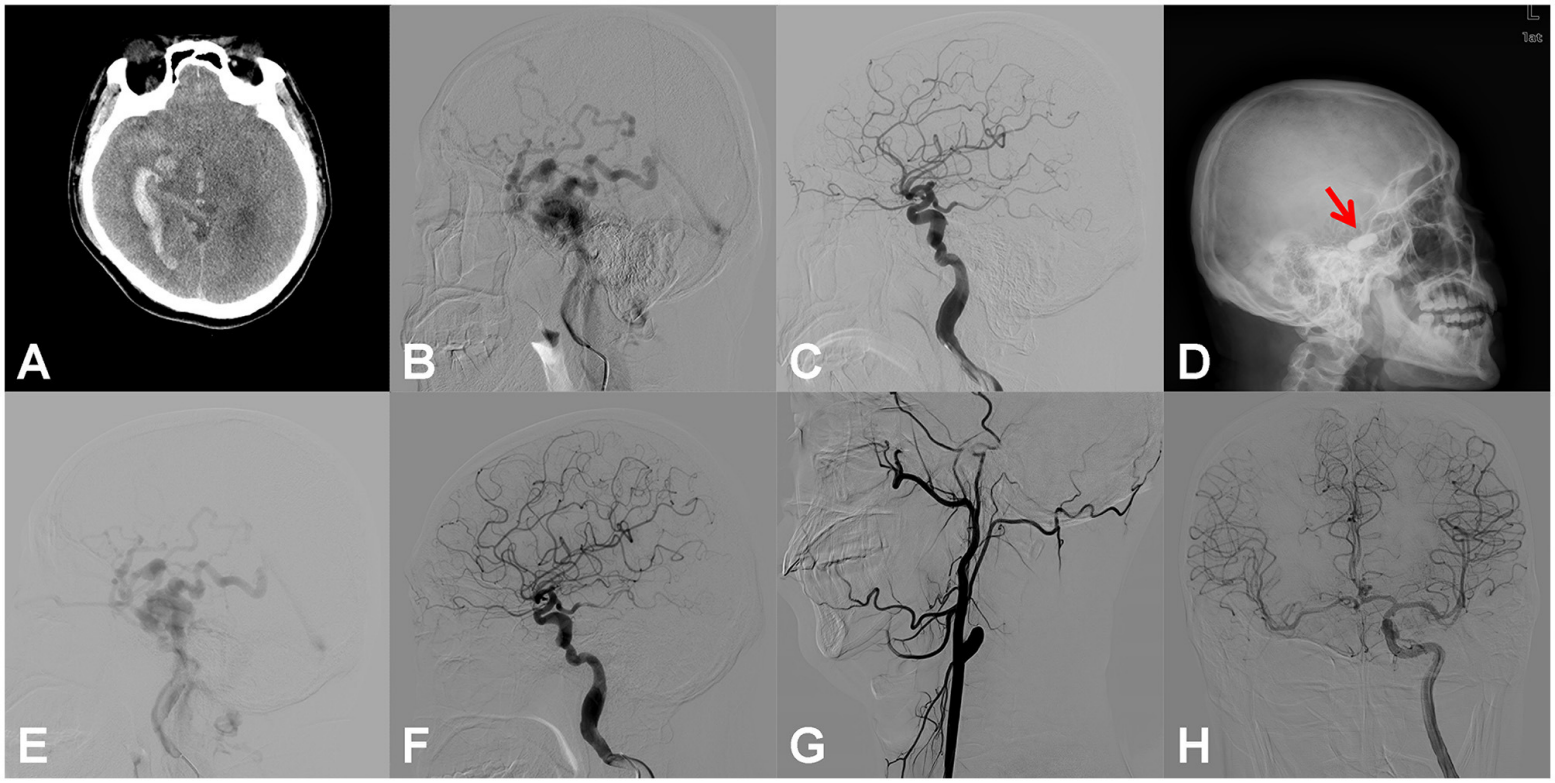
Right carotid cavernous fistula (CCF). An adult patient presented to the emergency room with an onset of severe headache for 6 h. Right temporal auscultation had a blowing murmur that was consistent with a heartbeat. **(A)** Emergency head computed tomography (CT) showed subarachnoid hemorrhage, right temporal lobe, and ventricular hematoma. **(B)** Digital subtraction angiography (DSA) of the right internal carotid artery (ICA) showed the CCF, with reflux drainage of cortical veins. **(C)** After the dilation of two detachable balloons (Goldbal2), the pathological shunt disappeared, and the cerebral blood flow recovered. **(D)** The patient suffered from sudden throbbing tinnitus on the left side 2 days after the endovascular procedure, and the head X-ray radiography revealed that only one balloon developed (red arrow). **(E)** Emergency DSA showed the recurrence of CCF. **(F)** With the introduction of another detachable balloon (Goldbal2) in the cavernous sinus, the pathological shunt disappeared angiographically and the patient recovered well without any neurological deficits. **(G)** DSA follow-up 6 months after the procedure showed complete occlusion of the lesional ICA. **(H)** DSA of the left ICA showed that the perfusion of the right hemisphere was compensated adequately by the anterior communicating artery.

**Figure 2:**
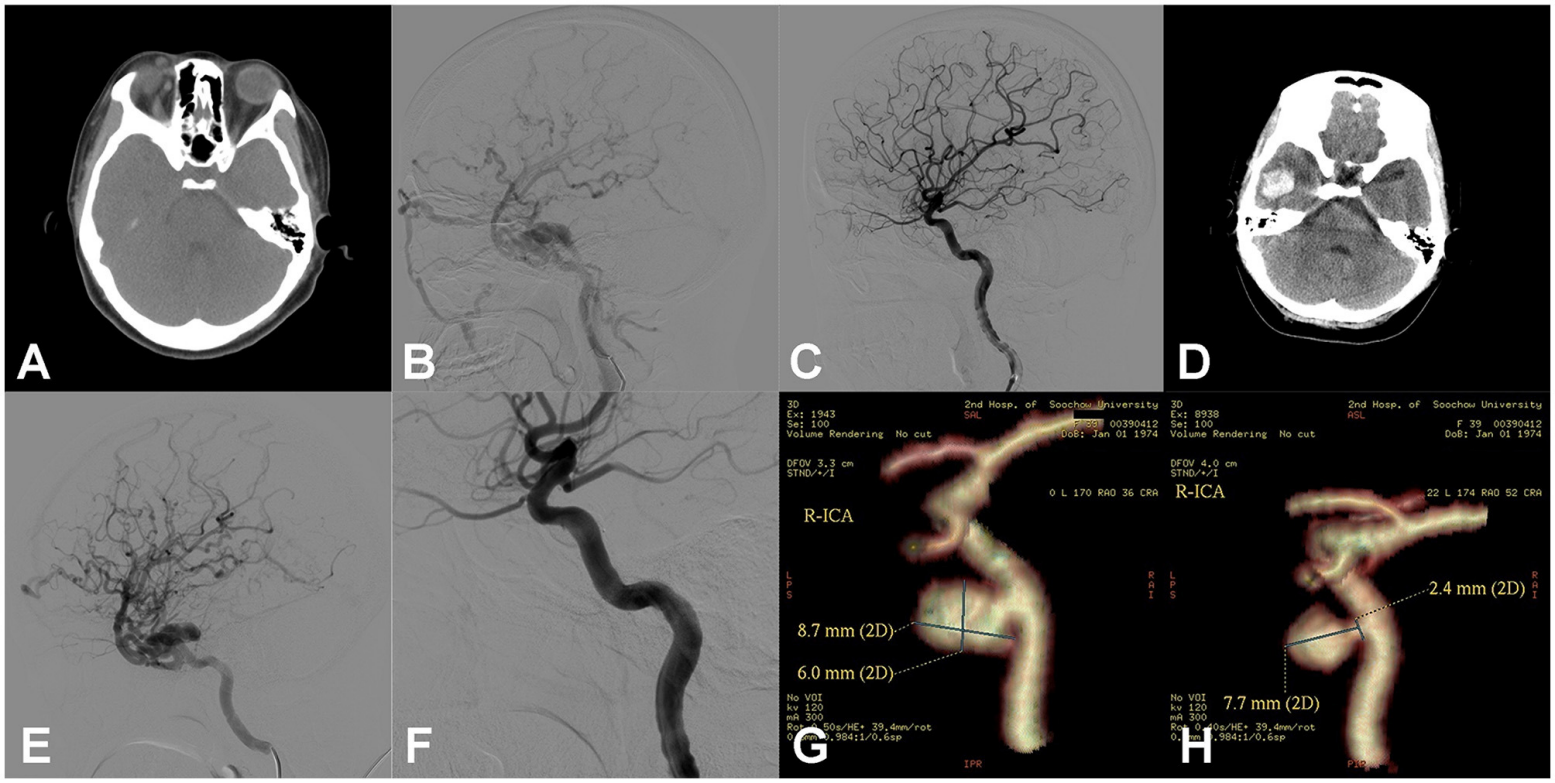
Right carotid cavernous fistula (CCF). An adult patient presented with right pulsing tinnitus after head trauma 1 month ago. Physical examination revealed a blowing murmur consistent with the heartbeat on the right temporal scalp, as well as a mild chemosis of the right side. **(A)** Head computed tomography (CT) showed the dilation of the right superior ophthalmic vein (SOV). **(B)** Digital subtraction angiography (DSA) of the right ICA showed the CCF with reflux drainage of cortical veins. **(C)** After dilation of one detachable balloon (Goldbal2), the pathological shunt disappeared and the cerebral blood flow recovered. The patient recovered well, and the symptoms of right chemosis and intracranial murmurs disappeared. **(D)** After 2 weeks, the patient presented to emergency with a sudden headache, and the head CT showed intracerebral hematoma of the right temporal lobe. The right temporal auscultation noted the recurrence of the blowing murmur. **(E)** Emergency DSA of the right ICA showed the recurrence of CCF and the reflux drainage of the cortical veins aggravated compared to previous images besides the undevelopment of SOV. **(F)** Another detachable balloon (Goldbal2) was introduced in the cavernous sinus, and the pathological shunt disappeared angiographically. The patient recovered well after the endovascular procedure with no neurological deficits. **(G)** After 3 months, CT angiography (CTA) showed the balloon had deflated and there was a consequent pseudoaneurysm in the cavernous sinus. The patient hesitated to receive another endovascular treatment. **(H)** In the 3-year follow-up, CTA showed the pseudoaneurysm was still present but smaller than before.

All patients were followed up for 3–10 years (average 75.0 months), and no recurrence of intracranial hemorrhage or CCF-related symptoms was noted. One patient who was treated with detachable balloons was found to have delayed spontaneous occlusion of the lesional ICA after 6 months, but the good compensation of the Willis circle resulted in an asymptomatic course (Figure 1). The lesional ICAs of the other four patients were intact. A pseudoaneurysm was noted by head CT angiography (CTA) in one patient who was treated with detachable balloons 3 months after the endovascular procedure, and the patient did not receive any further therapy. At the 3-year follow-up, a slight shrinkage of the pseudoaneurysm was noted in the patient's CTA image (Figure 2).

## 4. Discussion

### 4.1. Causes of ICH originating from CCF

CCF was first reported by Travers et al. in 1809. It can be divided into traumatic CCF and spontaneous CCF, according to the etiology. The former type is more common, accounting for ~75% of the cases (3). Barrow et al. classified CCFs into direct (Barrow A) and indirect types (Barrow B–D). The former has a direct shunt between the ICA and cavernous sinus, usually with high blood flow (4). All five patients in this study had a clear history of head trauma attributed to Barrow type A. The symptoms of CCF depend on the direction of venous drainage, and the most common symptom is caused by drainage into the superior ophthalmic vein (SOV), named the Dandy triple sign, which presents as pulsating exophthalmos, a murmur, and bulbar conjunctival edema (5). Drainage to the posterior superior and inferior petrosal sinuses is relatively rare and may cause cranial nerve palsy and hemiplegia. Directed upward drainage via cortical veins into the sagittal sinus and deep venous system is more dangerous (6). In ~9% of cases, increased venous pressure can generate reflux drainage of the cortical veins, which are prone to various forms of ICH and even fatal brain stem hemorrhage. If bulbous dilation of the draining veins is noted, the risk of ICH will be higher (7). In this study, all five patients had reflux drainage of the cortical veins, including the superior sagittal sinus in one patient, the straight sinus in one patient, and the superior sagittal and straight sinus in three patients. Three of them were complicated with a venous bulbous dilation. All of this evidence supports that cortical venous hypertension is a risk factor for CCF resulting in ICH. It has been reported that if anterior drainage through the SOV or posterior drainage through the superior and inferior petrosal sinuses is poor or absent, arterial hypertension will drain to the cerebral venous system through the sphenoid sinus or other channels, eventually causing ICH (8). Interestingly, one patient in this study had this extremely rare circumstance. This patient was diagnosed with CCF due to ocular symptoms, and half a month after the first endovascular procedure with detachable balloons, she was hospitalized again due to ICH. Comparing the DSA images of the two procedures, the anterior venous drainage disappeared due to SOV obstruction by a premature deflated balloon, but the cortical venous drainage caused by venous hypertension was much more obvious than before, which resulted in ICH of the right temporal lobe.

### 4.2. Selection of endovascular strategy

Generally, life-threatening CCF requires emergent management, and some of the high-risk factors for ICH include severe epistaxis, cortical venous reflux, and angiographic venous bulbous dilation. With the development of endovascular technology, the open surgical strategy for CCF has been almost eliminated. The choice of endovascular strategy should depend on the feeding artery, the draining vein, the blood flow velocity of the fistula, and the integrity of the Willis circle (9). Malan et al. classified traumatic CCFs into small, medium, and large CCFs based on the vascular structure, and this classification is helpful for the selection of different endovascular strategies (10).

Although there are increasing reports of the transvenous approach for the treatment of CCF, the transarterial approach is safer and simpler and is still suitable for the majority of such lesions. Many materials, including detachable balloons, detachable coils, Onyx glue, and covered stents, are available (11). From the perspective of economy and convenience, the detachable balloon is the preferred method, which can be completed under local anesthesia, and the operation is simple. Compared with detachable coil and Onyx glue embolization, detachable balloon embolization can be performed under local anesthesia, and the operation process is simpler (12). However, as in two of the patients in this study, after the first balloon embolization of case 2, the patient's right eye visual acuity decreased on the 10th day after the operation, and the CT examination showed bleeding. Further improvement of DSA showed that the ophthalmic vein pathway was blocked by the deflated balloon, the direction of venous drainage was changed, and a large number of cortical veins were countercurrent, resulting in bleeding. Therefore, detachable balloon embolization of the right internal carotid cavernous fistula was performed again at the same time. Postoperative cerebral angiography showed fistula occlusion. In case 4 after the first balloon embolization, on the 3rd day after the operation, the examination of the anterior and lateral cranial radiographs showed that only one of the two balloons remained. Combined with clinical practice, considering the possibility of balloon leakage, the leakage is broken again, and secondary interventional surgery is needed to re-block the leakage. DSA examination showed that the original embolization balloon disappeared, the fistula reopened, and the cortical vein drainage was obvious, so the detachable balloon embolization of the right internal carotid cavernous fistula was performed at the same time. Postoperative cerebral angiography showed fistula occlusion. Herein, we think recurrence caused by premature deflation is a major problem that needs to be considered. In addition, detachable balloons may eventually cause ICA occlusion and/or a cavernous sinus pseudoaneurysm, and these balloons have been less commonly used in the treatment of CCF (13). The detachable coil has good control ability, but there have been reports that mass stuffing in the cavernous sinus can cause compression of the cranial nerves and the possibility of aseptic inflammation, in addition to the high medical cost (14). In this study, two patients treated with a detachable coil had a small, cavernous sinus cavity that was adjacent to the fistula and were regarded as having cavernous aneurysms during the procedure. Some neurosurgeons advocated using Onyx glue, not only for its low cost but also to improve the safety of patients who had a higher risk of vascular injury due to their connective tissue disease (15). Another advantage of using Onyx glue is that collateral feeders, which are not visible on angiography, can be revealed during the progressive injection, and the use of Onyx glue results in improved cure rates. Therefore, the combination of Onyx glue with a detachable coil can not only retard shunt flow but also limit the diffusion of the Onyx glue, which ensures the therapeutic effect and reduces the filling degree of the Onyx glue in the cavernous sinus, and this is the preferred treatment at present (16). Covered stents and flow diverters (FDs) are also options for the treatment of CCF, but their use in patients with ICH remains controversial due to the need for subsequent dual antiplatelet therapy (17, 18).

## 5. Conclusion

The overall disability and mortality rate of CCF is low, and consequent ICH is relatively rare. However, it is a serious complication that may cause irreversible neurological deficits or a life-threatening prognosis. The recovery rate of endovascular procedures is 90–100%, the complication rate is low, and the mortality rate is < 1% (19–21). Therefore, individualized endovascular strategies should be carried out actively and in a timely manner for such patients.

## Data availability statement

The raw data supporting the conclusions of this article will be made available by the authors, without undue reservation.

## Ethics statement

Written informed consent was obtained from the individual(s) and/or minor(s)' legal guardian/next of kin for the publication of any potentially identifiable images or data included in this article.

## Author contributions

Conceptualization, supervision, and project administration: QZ. Methodology: QZ, A-LC, and C-GD. Software and visualization: Z-LL, A-LC, C-GD, D-HY, Y-HW, and YW. Validation and writing: Z-LL, A-LC, and YC. Investigation: A-LC and QZ. Resources and data curation: Z-LL and A-LC. All authors have read and agreed to the published version of the manuscript.
